# Endoscopic resection of GI stromal tumor using full-thickness resection device: tips and tricks

**DOI:** 10.1016/j.vgie.2022.10.006

**Published:** 2022-12-05

**Authors:** Ravi Jariwala, Laura Bratton, Ricardo Romero, John Evans, Janak Shah, Abdul Hamid El Chafic

**Affiliations:** Ochsner Medical Center, New Orleans, Louisiana

**Keywords:** FTRD, full-thickness resection device, GIST, gastrointestinal stromal tumor, SEL, subepithelial lesion

## Abstract

Video 1 R0 endoscopic resection of gastric GI stromal tumor using a dedicated gastroduodenal full-thickness resection device.

R0 endoscopic resection of gastric GI stromal tumor using a dedicated gastroduodenal full-thickness resection device.

## Background

Gastrointestinal stromal tumor (GIST) is the most common type of subepithelial lesion (SEL) in the stomach. Management of gastric GISTs varies by size. While all gastric GISTs ≥2 cm should be resected, the need to resect gastric GISTs <2 cm is still controversial given that surgical resection may be too aggressive for small, low-risk GISTs. On the other hand, evidence suggests that even <2-cm GISTs can metastasize.^1^ In fact, the Canadian guidelines suggest that even GISTs <1 cm should be excised because of the risk of metastases.^2^ Recent advances in endoscopic resection tools and techniques offer less-invasive options compared with surgery for small GISTs. Herein, we present a case of a <2-cm gastric GIST, endoscopically resected using a novel full-thickness resection device (FTRD) (Video 1, available online at www.giejournal.org).

## Case Presentation

**Figure 1:**
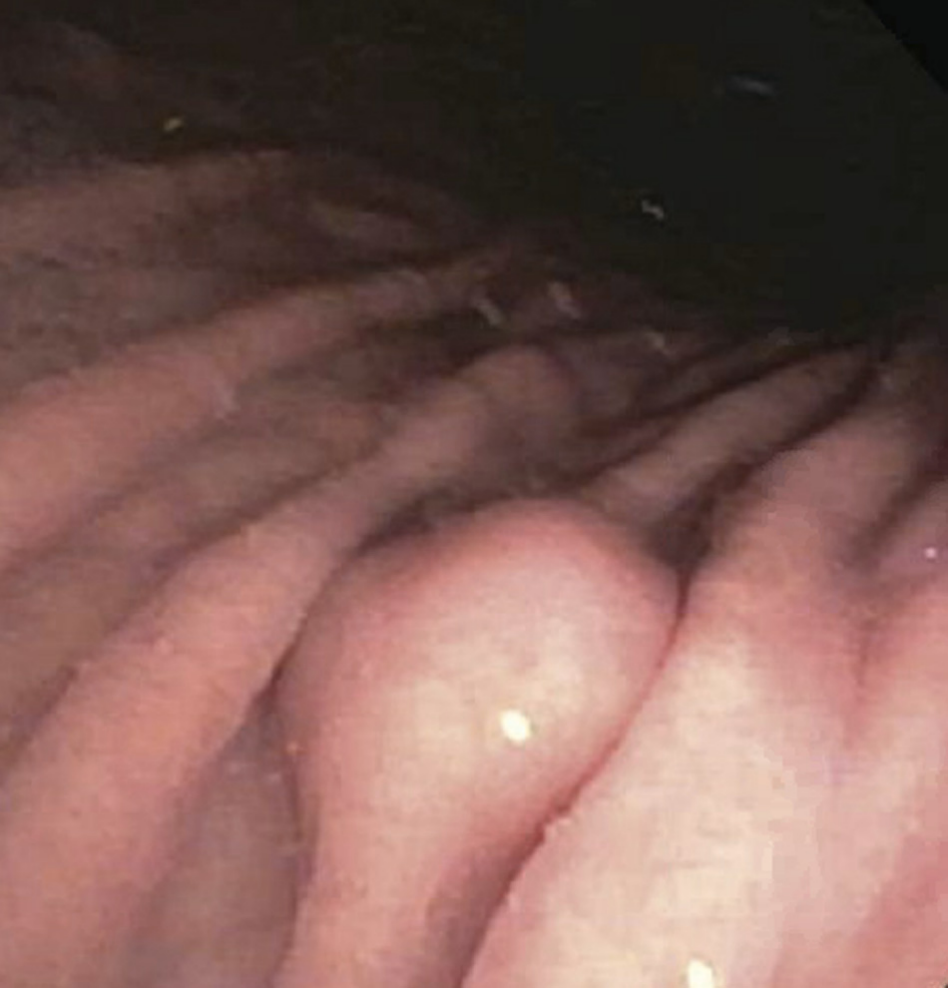
Gastric subepithelial lesion.

**Figure 2:**
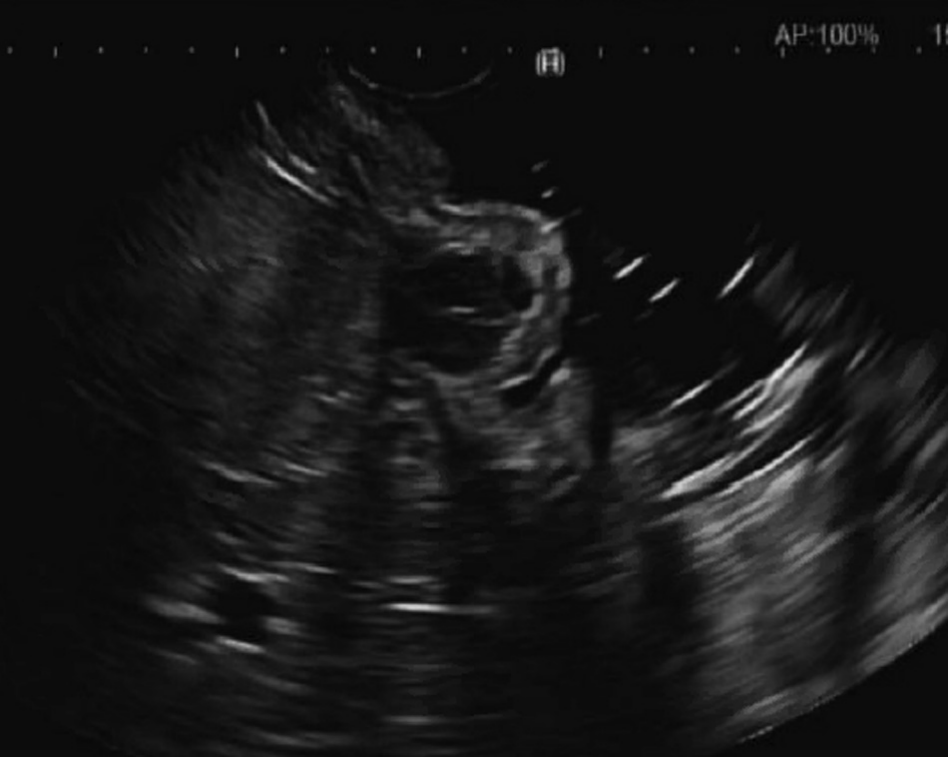
EUS showing a 10-mm hypoechoic lesion arising from the muscularis propria.

A 54-year-old woman with obesity and metabolic syndrome underwent EGD for a workup of chronic abdominal pain. The EGD examination was normal except for a 10-mm SEL that was found in the gastric body (Fig. 1). An endoscopic ultrasound showed a 10-mm hypoechoic lesion arising from the muscularis propria (Fig. 2). Fine-needle biopsy was then performed with cytology showing bland spindle cells and immunohistochemistry positive for CD117 and CD34 consistent with GIST. In our practice, we offer patients with <2-cm GISTs either yearly surveillance with EUS, surgical referral, or endoscopic resection with curative intent to avoid long-term surveillance with a R0 resection.^3^, ^4^, ^5^, ^6^ Management options were discussed with the patient, and the decision was made to proceed with endoscopic full-thickness resection to ensure negative margins using an FTRD.

## Procedure

A dedicated gastroduodenal FTRD was loaded on a dual-channel gastroscope to allow the usage of endoscopic devices in a separate channel from that of the FTRD thread, reducing chances of clip malfunction. The FTRD cap could not traverse the cricopharyngeus, so a dedicated insertion balloon was inflated to dilate the cricopharyngeus and fill up the FTRD applicator cap simultaneously for safer cap passage. The FTRD was then carefully slid distally to the proximal esophagus, after which the balloon was deflated and removed from the endoscope working channel.

**Figure 3:**
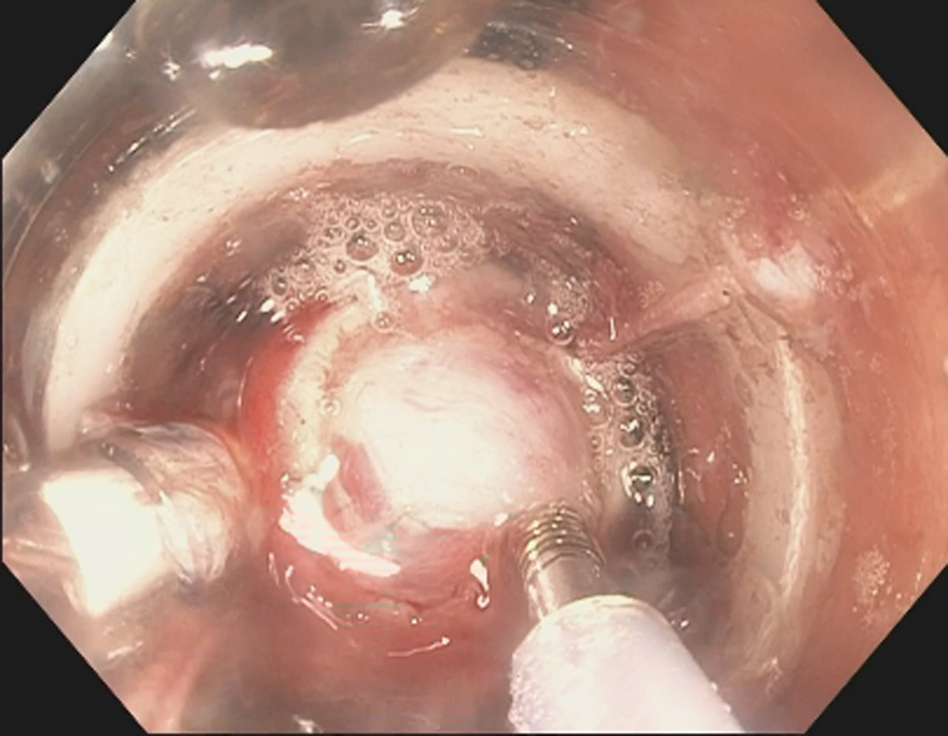
Lesion retracted into the full-thickness resection device cap via tissue helix.

**Figure 4:**
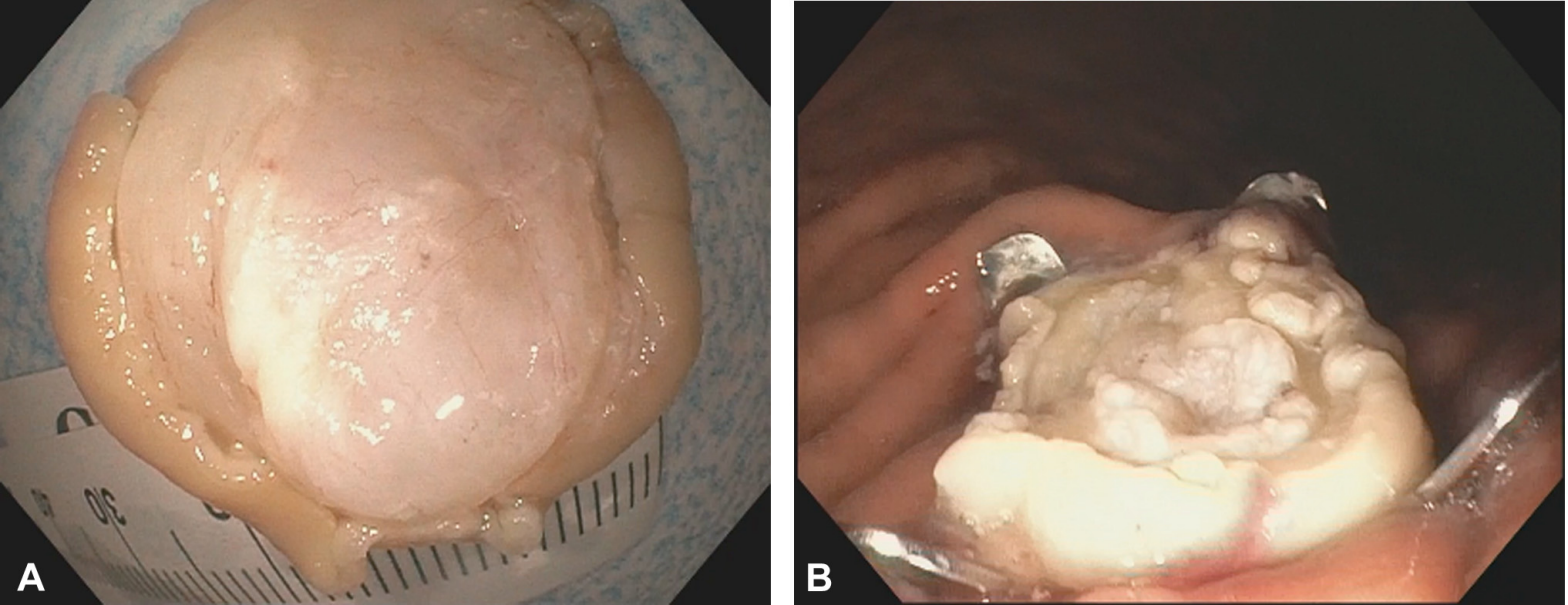
**A,** Deep margin of resected lesion. **B,** Resection site.

**Figure 5:**
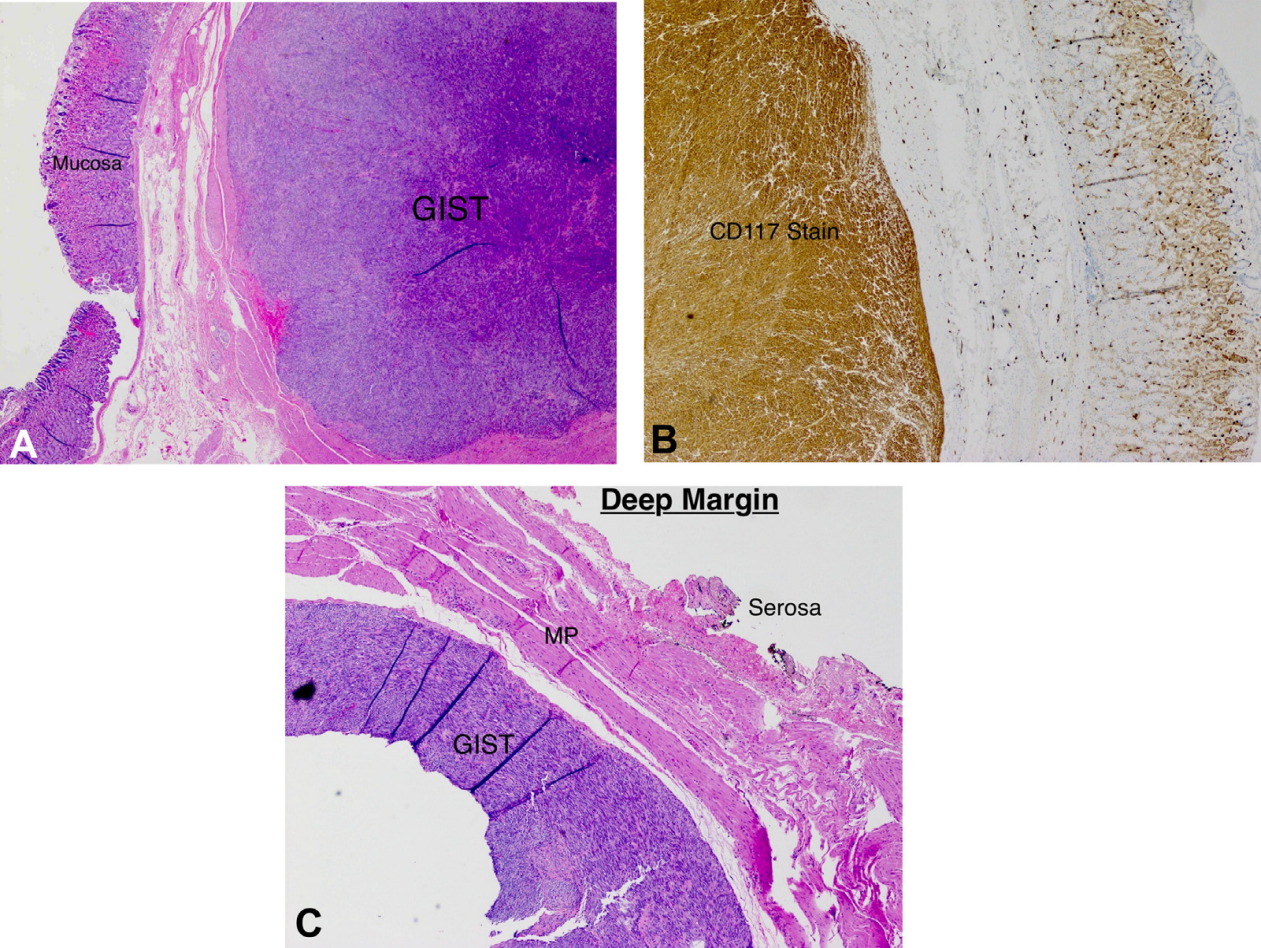
**A,** 2× lens magnification showing the GI stromal tumor with overlying submucosa and mucosa. **B,** 4× lens magnification showing strong and diffuse CD117 staining of the GIST. **C,** 4× lens magnification showing the deep margin with an R0 resection down to the serosa.

The endoscope with the application cap was directed over the previously marked SEL in the gastric body. For typical mucosal lesions resection via FTRD system, a grasper is used to mobilize the lesion. Mobilizing the SEL into the cap by the grasper was attempted; however, only the overlying mucosa was grasped and then detached, resulting in failure to retract the lesion. We then decided to better expose the SEL by resecting the overlying mucosa with a snare. After exposure of the SEL, we decided to grasp the lesion with a tissue helix. We believe that the tissue helix offers deeper penetration to capture the SEL than the grasper or the tri-prong anchor. The helix was advanced to the exposed SEL, and while using forward pressure, the tissue helix knob was rotated clockwise so the helix would penetrate and capture the SEL. The helix and the captured SEL were gently and steadily retracted into the application cap (Fig. 3). The FTRD clip was applied with simultaneous en bloc resection using the premounted electrosurgical snare. The resected lesion was retrieved, and the resection site was inspected showing no immediate adverse event (Fig. 4). The patient was discharged home on the same day as the procedure. The patient was contacted by phone 4 weeks later for follow-up, and no delayed adverse events were reported. Final pathology confirmed a GIST with R0 resection (Fig. 5).

## Conclusion

Dedicated gastroduodenal FTRD has become available recently, offering an endoscopic alternative for resecting gastroduodenal SELs. Gastric GISTs are known to be difficult to mobilize and retract into the FTRD cap secondary to the thick gastric wall and the hard nature of GISTs. Herein, we present a novel technique by which we exposed the SEL and then used a tissue helix to better retract the GIST into the FTRD system. Achieving R0 resection demonstrates the efficacy of this technique.

## Disclosure

*Dr Shah is a consultant with Olympus and BSCI. All other authors disclosed no financial disclosures.*

## Supplementary data

Video 1 R0 endoscopic resection of gastric GI stromal tumor using a dedicated gastroduodenal full-thickness resection device.
